# Robot-assisted radical prostatectomy in the Middle East: A report on the perioperative outcomes from a tertiary care centre in Lebanon

**DOI:** 10.1080/2090598X.2020.1814184

**Published:** 2020-08-26

**Authors:** Muhieddine Labban, Muhammad Bulbul, Wassim Wazzan, Raja Khauli, Albert El Hajj

**Affiliations:** Division of Urology, Department of Surgery, American University of Beirut Medical Center (AUBMC), Beirut, Lebanon

**Keywords:** Middle East, perioperative outcomes, prostate cancer, robotics, robot-assisted radical prostatectomy

## Abstract

**Objective:**

To report on the surgical, oncological and early functional outcomes of robot-assisted radical prostatectomy (RARP) at our tertiary care centre, as there is a scarcity of reports on outcomes of robotic surgery from the Middle East.

**Patients and methods:**

We reviewed the electronic health records for patients undergoing RARP between 2013 and 2019 at the American University of Beirut Medical Center. We collected patients’ demographics and preoperative oncological factors including prostate-specific antigen (PSA), clinical oncological stage, and World Health Organization (WHO) grade. PSA persistence, biochemical recurrence (BCR) and positive surgical margin (PSM) were reported. Complications were categorised by Clavien–Dindo grade. Moreover, the postoperative oncological outcomes including the rates of adjuvant and salvage androgen-deprivation therapy (ADT) and external-beam radiation therapy (EBRT), chemotherapy, and metastasis were reported. Additionally continence and potency results were retrieved.

**Results:**

For the designated period, 250 patients underwent RARP of which 182 (72.8%) underwent lymph node dissection. The median (interquartile range) anaesthesia time was 330 (285–371) min and the estimated blood loss was 200 (200–300) mL. The overall complication rate was 8%, with 2% Clavien–Dindo Grade III–IV complications. The PSM and BCR rates were 21.6% and 6.4%, respectively. Adjuvant ADT and EBRT was administered to 7.2% of the patients. Functional data was available for 112 patients. Continence was 68%, 82% and 97% of the patients at 3, 6 and 12 months, respectively. For 65 patients who had bilateral nerve sparing potency was 37%, 60% and 83% at 3, 6 and 12 months, respectively.

**Conclusion:**

This is the largest RARP series from the Middle East. The surgical, oncological and functional outcomes are consistent with those published in the literature. This confirms the safety and efficacy of applying robotic technology in our region during the implementation phase.

**Abbreviations:** ADT: androgen-deprivation therapy; AJCC: American Joint Committee on Cancer; AUBMC: American University of Beirut Medical Center; BCR: biochemical recurrence; CPT: Current Procedural Terminology; EBRT external beam radiation therapy; IQR, interquartile ranges; LOS: length of stay; PLND: pelvic lymph node dissection; PSM: positive surgical margin; (O)(RA)RP, (open) (robot-assisted) radical prostatectomy

## Introduction

Robotic surgery was introduced to the realm of minimally invasive urological surgery at the beginning of the 21st century with the first robot-assisted radical prostatectomy (RARP) performed in 2001 [1]. In contrast to laparoscopy, robot-assisted surgeries allow three-dimensional visualisation of the operative field, offer 7 degrees of freedom of wrist movement, and eliminate the challenges of the fulcrum effect [2]. Despite the technological advantages, open RP (ORP) and RARP demonstrate comparable functional and oncological outcomes [3]. Yet, RARP is associated with reduced intraoperative adverse events, decreased blood loss, and a shorter postoperative hospital stay [4].

The Intuitive Surgical’s (Sunnyvale, CA, USA) da Vinci robot dominates the market with >4000 units installed worldwide in 2017 and is featured in >10 000 peer-reviewed publications [5]. The vast majority of these robots (82%) are found in North America and Europe; while only 44 robots are installed in the Middle East, most of them (19 out of 44; 43%) concentrated in Saudi Arabia [6]. On the other hand, Lebanon owns three da Vinci robots located in private and academic institutions. The first RARP in the region was performed in July 2013 at the American University of Beirut Medical Center (AUBMC), 10 years after the first procedure in Saudi Arabia [7]. Data collected from the local distributor of the da Vinci robot revealed a 3.4-fold increase in urological robotic surgeries in Saudi Arabia from 2010 to 2017 [6]. However, the rate of adoption of the da Vinci robots in the Middle East was not met by a parallel research output tackling surgical outcomes and quality improvement in urology. In fact, a review of the literature revealed three urological robotic surgery series from the Middle East: one from Egypt published in 2004, another from Saudi Arabia published in 2012, and a single-surgeon RARP series from Kuwait (2020) [8–10]. Only the last series by Aldousari *et al*. [10] reported on surgical and functional outcomes.

After the introduction of robotics at the AUBMC in 2013, the total number of prostatectomies performed increased by 8% with a congruent drop witnessed in ORP (11.5%). The initial cautious increase rate of RARP was attributed to its elevated cost, reluctance of patients to opt for a new technique, and reduced rates of referrals [7]. Due to the scarcity of robotic surgery reports from the Middle East, we would like to present our RARP series and explore the perioperative and long-term medical and oncological outcomes of patients who received treatment at our tertiary care centre.

## Patients and methods

### Patient selection

After receiving Institutional Review Board approval, a robotic surgical database was initiated. The institution’s electronic health record was reviewed for the Current Procedural Terminology (CPT) code for RARP (CPT 55866) for patients diagnosed with prostate cancer between July 2013 and July 2019 at the AUBMC, Lebanon. Prior to the introduction of the da Vinci robot to our institution, all procedures were performed by open technique. Currently, all RARP procedures are either fully performed or proctored by a fellowship-trained urological oncologist (A.E.H.). In addition to the main operator, the series included three additional surgeons.

### Surgical technique

Pneumoperitoneum is achieved via a midline supra-umbilical incision deepened sharply to the rectus fascia. Then, the rectus fascia is opened and access to the peritoneum is done using the Hassan open approach. The camera is introduced through this trocar. Four other trocars are inserted under laparoscopic vision: two 8-mm robotic trocars in the right quadrant at the same level as the camera port, an 8-mm robotic trocar superior and medial to the left iliac crest and an 11-mm assistant trocar in the left upper quadrant.

In case of bilateral extended pelvic lymph node dissection (PLND), access to the peritoneum is achieved lateral to the medial umbilical ligament. The lateral limit of the dissection is the genitofemoral nerve and the medial limit is the obturator nerve, while the distal limit is the inguinal ligament and the proximal limit is the aortic bifurcation.

The RARP is started by first reflecting the bladder off the anterior abdominal wall. After the dissection of the space of Retzius, the prostate is identified and cleaned off. Then, the endopelvic fascia is carefully dissected and opened in order to dissect the space between the prostate and the levator ani muscle. This step is performed bilaterally. After dividing the anterior bladder neck at the prostate-vesical junction, the Foley catheter is identified within the bladder and grabbed with the fourth robotic arm to lift the prostate. Consequently, the posterior bladder neck is divided carefully and the dissection is carried through the posterior layers of the bladder wall. The vas deferens and seminal vesicles are then identified on either side and carefully dissected free. Next, Denonvilliers’ fascia is divided at the plane between the posterior surface of the prostate and the rectum could be developed. The prostatic pedicles are then divided and sealed using Hem-o-lok clips. On a case-by-case basis, intrafascial or interfascial nerve sparing could be performed. At the apex of the prostate, the dorsal vein is identified and a V-Loc suture is applied to the dorsal vein in order to tie it off and suspended to the pubic bone. The dorsal vein complex and the apex of the prostate should be divided carefully and then the rectourethralis muscle is divided sharply. As such, the prostate specimen is completely freed and placed in an EndoCatch bag.

The anastomosis between the urethra and the bladder neck is completed posteriorly first. Then, anterior anastomosis is completed using Stratafix barbed suture. A silicone catheter is placed into the bladder after the anastomosis is made and the bladder filled to check for any fluid extravasation.

### Data collection

Patients’ demographics and preoperative oncological factors including serum PSA level, clinical oncological stage estimated on DRE, and pathological WHO grade obtained from TRUS core biopsies were recorded. Neoadjuvant treatment (GnRH agonist), Goserelin 10.8 mg, was offered for 3 months in some patients to improve local disease control or down staging. We defined PSA persistence as a PSA level of >0.1 ng/mL and biochemical recurrence (BCR; PSA failure) as a postoperative PSA level of ≥0.2 ng/mL, with a second confirmatory level exceeding 0.2 ng/mL. Moreover, patients’ stages were assigned in line with the American Joint Committee on Cancer (AJCC). We also added the patients’ anaesthesia time, as the patient-specific operative time was not recorded. Using the institution’s pathology report, information on WHO grade, prostate weight, and tumour characteristics (including information on extraprostatic extension, perineural invasion, and lymphovascular, seminal vesicle, or surgical margin involvement) were documented. We also reported on lymph nodes involved in patients who underwent PLND. All patients underwent the same technique for lymphadenectomy entailing an extended PLND by excising all fatty tissue up to the common iliac bifurcation and deep to the obturator fossa. We also included the lymph node yield or the median number of lymph node retrieved and percentage of tumour involved lymph nodes included the ratio of positive retrieved lymph nodes to the total number of lymph nodes dissected. Functional outcomes including urinary continence and potency were retrieved from follow-up notes at 3, 6 and 12 months. Continence was defined as a strict no pads use and potency was defined as the ability to penetrate.

Postoperative surgical outcomes encompassing any 30-day postoperative adverse event categorised by Clavien–Dindo Grade and patients’ length of stay (LOS) were entered. We used the revised Clavien–Dindo grading system to classify the surgical complications thereby permitting institutional outcome comparison. Additionally, postoperative oncological outcomes capturing the rates of adjuvant and salvage androgen-deprivation therapy (ADT) and external beam radiation therapy (EBRT), chemotherapy, and metastasis were reported. Treatment with ADT and EBRT combination was offered as either in adjuvant form due to surgical margin involvement or PSA persistence, or as salvage therapy in the case of PSA failure.

### Analysis

Medians and interquartile ranges (IQRs) were reported for continuous variables and counts and percentages were used for categorical variables. The Statistical Package for the Social Sciences (SPSS®) for Windows, version 24 (IBM Corp., Armonk, NY, USA) was used to report the results.

## Results

**Figure 1.**
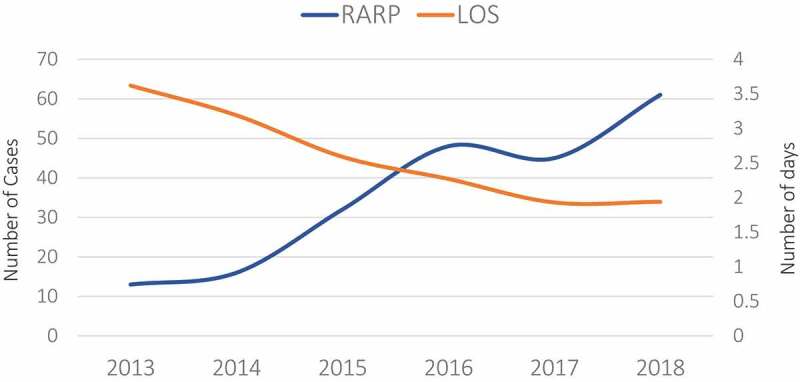
The trend of the load of RARP cases and the length of hospital stay (days) from 2013 to 2018

For the designated period, 250 patients underwent RARP of which 182 (72.8%) underwent extended PLND (Table 1). The median (IQR) patient age was 64 (58–69) years and 33.6% of the patients were obese. On presentation, 97 patients (42.9%) had a WHO Grade ≥3, 49 patients (19.6%) were staged as ≥T2c on DRE, and the median (IQR) preoperative PSA level was 7.2 (5.0–10.0) ng/mL. Additionally, the median anaesthesia time was 330 (285–371) min. Figure 1 depicts the number of cases performed and the average LOS (days) as a function of time (years). The number of RARPs performed doubled between 2015 and 2019 (32 vs 60 cases) and the mean (SD) LOS considerably dropped from 3.62 (1.39) days in 2013 to 1.95 (0.75) days in 2018 (*P* < 0.001).

**Table 1.** Demographics of patients undergoing RARP (*N* = 250)

Variable	Value
Age, years, median (IQR)	64 (58–69)
BMI ≥30 kg/m^2^, *n* (%)	84 (33.6)
Anaesthesia time, min, median (IQR)	330 (285–371)
Estimated blood loss, mL,, median (IQR)	200 (200–300)
Preoperative PSA level, ng/mL, median (IQR)	7.2 (5–10)
Lymphadenectomy, *n* (%)	182 (72.8)
Neoadjuvant treatment, *n* (%)	8 (3.2)
Grade on TRUS, *n* (%)	
≤2	142 (57.0)
≥3	107 (43.0)
Clinical Stage on DRE, *n* (%)	
T1c	87 (34.8)
T2a	72 (28.8)
T2b	32 (12.8)
T2c	21 (8.4)
T3a	22 (8.8)
T3b	6 (2.4)

On pathological evaluation, 123 patients (54.7%) were assigned to a WHO Grade ≥3 (Table 2). The rates of pathological upgrading and downgrading were 38.1% and 16.9%, respectively. Most of the pathological upgrading (81.4%) and the majority of the pathological downgrading (84.2%) were in the order of one WHO grade. The median (IQR) prostate specimen weight was 50 (40–65) g. The pathologists reported extraprostatic extension, seminal vesicle invasion, and lymphovascular involvement in 25.6%, 12.0%, and 7.2% of the patients, respectively. Surgical margin tumour involvement was documented in 21.6% of patients; yet, in a third of the cases (32.3%) the PSM length did not exceed 3 mm. The proportion of tumour involved surgical margins for pT2, and pT3 was 17.4%, and 30.0%, respectively. Additionally, tumour lymph node involvement was found in 26 patients (14.4%). The median (IQR) yield of lymph nodes dissected was 14 (10–20) and the mean (SD) percentage of tumour-involved lymph nodes was 2.0 (9.1)%. Data on functional outcome was available for 112 patients (44.8%). Urinary continence was achieved in 68%, 82% and 97% of patients at 3, 6 and 12 months, respectively. From this group of patients 65 (58%) had bilateral nerve sparing. Potency was achieved in 37%, 60% and 83% at 3, 6 and 12 months, respectively. We reported on the 30-day surgical outcomes categorised by Clavien–Dindo grade. In total, we recorded six Grade I (2.4%), 17 Grade II (6.8%), one Grade IIIa (0.4%), and four Grade IVa (1.6%) (Table 3). None of the patients underwent re-operation (Grade IIIb) or had multiple organ dysfunction (Grade IVb). Most of the adverse events were classified as Grade II and were attributed mostly to UTIs (seven patients; 2.8%) treated with antibiotics (Table 3). The rates of transient anastomotic leaks were minimal (two patients; 0.8%) and were managed by extended urethral catheterisation. Similarly, the median (IQR) estimated blood loss was 300 (200–300) mL and only three patients (1.2%) received transfusion of blood products (Tables 1 and 3). In our cohort, we only had one of each of the following complications: hospital-acquired pneumonia, *Clostridium difficile* infection, and pulmonary embolus. Moreover, none of the patients were re-operated in the 30-day postoperative period. In this series, three patients (1.2%) developed sepsis (Grade IVa) at 4–12 days postoperatively necessitating admission to the intensive care unit. Adjuvant ADT with EBRT was administered to 18 patients (7.2%) for PSA persistence (five patients, 2%), PSMs (four patients, 1.6%), or both (nine patients, 3.6%). Salvage EBRT was administered to 16 patients (6.4%) for BCR. Metastasis developed in 15 patients (6.0%) and four patients (1.6%) progressed to castrate-resistant prostate cancer and received chemotherapy. Only one patient (0.4%) died due to a second primary tumour.

**Table 2.** Oncological and pathological variables of patients undergoing RARP (*N* = 250)

Variable	Value
Pathological Grade, *n* (%)	
≤2	112 (45.3)
≥3	135 (54.7)
Upgrading rate^a^, *n* (%)	99 (40.2)
Downgrading rate^b^, *n* (%)	41 (16.7)
Prostate weight on pathology, g, median (IQR)	50 (40–65)
Extraprostatic extension, *n* (%)	64 (25.6)
Seminal vesicle involvement, *n* (%)	30 (12.0)
Perineural invasion, *n* (%)	178 (71.2)
Lymphovascular involvement, *n* (%)	18 (7.2)
Surgical margin involvement, *n* (%)	54 (21.6)
Lymph node involvement, *n* (%)	26 (14.3)
AJCC Stage, *n* (%)	
1	27 (10.8)
2	134 (53.6)
3	60 (24.0)
4	24 (10.4)

**Table 3.** The 30-day medically stratified by Clavien–Dindo Grade and oncological outcomes following RARP

Surgical outcomes		
Variable	*N* (%)	Management
Clavien–Dindo Grade I		
Scrotal haematoma	2 (0.8)	Scrotal elevation
Allergic exanthema	1 (0.4)	Antihistamine
Transient anastomosis leak	2 (0.8)	Extended urethral catheterisation
Transient elevation of creatinine	1 (0.4)	Hydration
Clavien–Dindo Grade II		
Ileus	1 (0.4)	Conservative
UTI	7 (2.8)	Antibiotics
Hospital-acquired pneumonia	1 (0.4)	Antibiotics
*Clostridium Difficile*	1 (0.4)	Antibiotics
Surgical-site Infection	2 (0.8)	Antibiotics
Blood transfusion	3 (1.2)	Blood products
Lymphoedema	2 (0.8)	Limb elevation
Clavien–Dindo Grade IIIa		
Intra-abdominal abscess	1 (0.4)	Percutaneous drainage
Clavien–Dindo Grade IIIb		
Nil	–	–
Clavien–Dindo Grade IVa		
Sepsis	3 (1.2)	Transfer to surgical intensive care unit and antibiotics administration
Pulmonary embolus	1 (0.4)	Anticoagulation
Clavien–Dindo Grade IVb		
Nil	–	–
Clavien–Dindo Grade V		
Nil	–	–
Oncological Outcomes		
Adjuvant ADT + EBRT	18 (7.2)	
Salvage ADT + EBRT	16 (6.4)	
Metastasis	15 (6.0)	
Chemotherapy	4 (1.6)	
Mortality	1 (0.4)	

## Discussion

In the present study, we report our series of patients who underwent RARP with or without bilateral PLND in a Middle Eastern tertiary care centre with specific focus on the surgical complications classified by Clavien–Dindo grade and oncological outcomes. More than 1500 ORPs were performed at our institution prior to the introduction of the robot. At the introduction of the robot, A.E.H., the principal operator of the series, had performed >600 robot-assisted surgeries for the prostate, kidneys, and bladder. Three other surgeons with a large experience in open surgery contributed to the present series.

Patel *et al*. [11] conducted a survey of 1000 patients seeking robotic surgery and found that most of patients sought it for decreased morbidity. The acquisition of the da Vinci robot to our institution clearly translated into an increase in the number of procedures performed presumably because patients who may have been reluctant to opt for surgery reconsidered their options, especially with the benefits of reduced blood loss, decreased perioperative pain, and limited hospital stay [11]. Although the average LOS was elevated in 2013; starting from 2017, most of the patients were discharged either on postoperative day 1 or day 2. The latest practice is consistent with the practice in the United States [11].

We observed 25 complications in 20 patients (8.0%); in comparison, in a large series comprising 2500 cases performed by a single surgeon, Coelho *et al*. [12] reported adverse events in 5% of the patients. Although we had an equivalent Grade I complication rate (2.24% vs 2.4% in our present series), we had more Grade II complications (6.8% vs 1.8% in the Coelho *et al*. [12] series). In addition, while ileus was the largest contributor to Grade II complications in the Coelho *et al*. [12] series, most of the Grade II complications in our present series were attributed to UTIs. Furthermore, similar to our present findings, the Grade IVa complications were caused by pulmonary embolism [12]. The discrepancy in the complication rates is explained by the size of our series [12]. Effectively, Badani *et al*. [13] reported on 2766 cases performed by different urologists and found a comparable rate of complications (12.2%). Furthermore, in contrast to Coelho *et al*. [12], where >80% of the complications were either Grade I or II, the Grade I and II complications in our present series represented 69.7% of all adverse events. However, our numbers were congruent to the smaller Murphy *et al*. [14] series of 400 cases, where 66.7% of the complications were Grade I and II. This affirms that the severity of complications decreases along a surgeon’s learning curve.

Surgeons often rely on the status of the surgical margins to predict oncological outcomes such as BCR and the need for secondary treatment. Although RARP helps surgeons achieve a lower PSM rate in comparison to its open counterpart, the rate of PSM decreases along a surgeon’s learning curve [11]. In the Patel *et al*. [11] single surgeon series of 1500 patients, the authors reported a PSM rate of 12% for the first 300 cases, but reached nadir (2%) after the 1200th case. In our present series, we also observed a drop in the rate of PSM from 38.5% to 20.5%. Besides, higher overall PSM rates are acknowledged in multi-institutional series and in studies involving multiple surgeons. For instance, Menon *et al*. [15] reported on the outcomes of 2562 RARPs performed by several surgeons and recorded a PSM rate of 13%. Similarly, a large multi-institutional series by Patel *et al*. [16] incorporating 8418 patients documented a congruent PSM rate (15.7%). As clinical stage is a strong predictor of PSM, it is worth exploring the stage-specific PSM rates to further allow comparison of results. In the latter series, the stage-specific PSM rate was 9.45%, 37.2% for pT2 and pT3 disease, respectively [16]. In comparison, our present PSM rate was higher for pT2 (17.4%), but the rate for pT3 was lower (30.0%). Furthermore, Ploussard *et al*. [17] from France published on their first experience with RARP. The authors compared the PSM rate between 1377 patients undergoing laparoscopic RP and 1009 patients undergoing RARP. The rate of PSM was higher in RARP (31.3%) than in laparoscopic-assisted RP (26.6%). Their stage-specific PSM rate for pT3 disease was particularly very elevated (47.4% vs 30.0% in our present cohort). Despite the limited size of our present series and our relatively recent experience in RARP at our institution, we achieved comparable outcomes to other large multi-institutional studies. False-positive tumour involvement of the surgical margins could also justify our mildly inflated PSM rate. This occurs in cases of accidental capsular laceration where sub-capsular cancerous cells become exposed [18]. Furthermore, a third of the surgical margins in our series were ≤3 mm and often focal in nature. As a limited PSM size would neither impact local recurrence or PSA failure, we did not expect an elevated BCR [19].

In our present cohort, the median (IQR) follow-up was 26.5 (12–42) months and the BCR rate was 6.4% of the patients. Subsequently, those patients received ADT and EBRT. Menon *et* *al.* [20] published on the biochemical recurrence of 1384 patients following RARP. BCR-free rates of 95.1%, 90.6%, and 86.6% at 1, 3, and 5 years respectively were recorded. Thus, our present numbers fall well on their curve. In a smaller series from Thomas Jefferson University including 439 patients treated with RARP by a single surgeon, the authors noted a BCR rate of 7.7% [21]. However, their population had a smaller proportion of WHO Grade ≥4 (9.3% vs 19.9% in our present series) and Stage ≥3 (23.0% vs. 34.4% in our present series) [21]. This indicates that patients in our region either present late in the course of the disease or inherently have a more aggressive biology. Moreover, as our present cohort is the first to undergo RARP at our institution, we expect a decrease in the BCR rate along the surgeons’ learning curve. This was well illustrated by a large single surgeon observational data from Australia, which showed that the risk of BCR among the first RARP is three-times that of ORP [22]. Nevertheless, a BCR-free survival superior to the open technique is achievable after a relatively long learning curve of 226 cases [22].

Although the functional outcomes were only available in 44.8% of our present patients due to the short follow-up period, continence and potency outcomes were consistent with the published literature [4,11,15].

At a regional level, the Aldousari *et al*. [10] single-surgeon RARP series of 65 patients is the only series published from the Middle East. Our present population had similar median preoperative PSA levels and prostate weight, but had a higher pathological WHO Grade ≥3 (35% vs 54.7% in our present series). Although the two series had similar lymphadenectomy rates, the Aldousari *et al*. [10] series had twice the rate of adjuvant therapy (14% vs 7.2% in our present series). Similarly, the BCR rate was also higher in their series (10% vs 6.4% in our present series).

### Limitations

Although our present series is limited in number, to our knowledge it is the largest from the Middle East reporting on robotic surgical outcomes. Our data lack information on patient’s comorbidities or frailty. Moreover, future reports should shed light on the functional outcomes using standardised questionnaires on the incontinence rate, quality of life, sexual and erectile outcomes when enough follow-up time is reached for the majority of the patients.

## Conclusion

We report on a RARP series from a Middle Eastern tertiary care centre. These results are consistent with the published literature and confirm the safety and efficacy of this approach. This study also confirms the safety and efficacy of applying robotic technology in our region during the implementation phase.
